# Identification of a novel interaction of FUS and syntaphilin may explain synaptic and mitochondrial abnormalities caused by ALS mutations

**DOI:** 10.1038/s41598-021-93189-6

**Published:** 2021-06-30

**Authors:** Shaakir Salam, Sara Tacconelli, Bradley N. Smith, Jacqueline C. Mitchell, Elizabeth Glennon, Nikolas Nikolaou, Corinne Houart, Caroline Vance

**Affiliations:** 1 Department of Basic and Clinical Neuroscience, Institute of Psychiatry, Psychology and Neuroscience, King’s College London, Denmark Hill, London, SE5 8AF UK; 2 Centre for Developmental Neurobiology and MRC CNDD, Institute of Psychiatry, Psychology and Neuroscience, Guy’s Campus, King’s College London, London, SE1 1UL UK; 3 Department of Biology & Biochemistry, University of Bath, Claverton Down, Bath, BA2 7AY UK

**Keywords:** Cellular neuroscience, Diseases of the nervous system, Molecular neuroscience, Motor control

## Abstract

Aberrantly expressed fused in sarcoma (FUS) is a hallmark of FUS-related amyotrophic lateral sclerosis (ALS) and frontotemporal dementia (FTD). Wildtype FUS localises to synapses and interacts with mitochondrial proteins while mutations have been shown to cause to pathological changes affecting mitochondria, synapses and the neuromuscular junction (NMJ). This indicates a crucial physiological role for FUS in regulating synaptic and mitochondrial function that is currently poorly understood. In this paper we provide evidence that mislocalised cytoplasmic FUS causes mitochondrial and synaptic changes and that FUS plays a vital role in maintaining neuronal health in vitro and in vivo. Overexpressing mutant FUS altered synaptic numbers and neuronal complexity in both primary neurons and zebrafish models. The degree to which FUS was mislocalised led to differences in the synaptic changes which was mirrored by changes in mitochondrial numbers and transport. Furthermore, we showed that FUS co-localises with the mitochondrial tethering protein Syntaphilin (SNPH), and that mutations in FUS affect this relationship. Finally, we demonstrated mutant FUS led to changes in global protein translation. This localisation between FUS and SNPH could explain the synaptic and mitochondrial defects observed leading to global protein translation defects. Importantly, our results support the ‘gain-of-function’ hypothesis for disease pathogenesis in FUS-related ALS.

## Introduction

Amyotrophic Lateral Sclerosis (ALS) and Frontotemporal Dementia (FTD) are both fatal neurodegenerative diseases which show a large degree of clinical, pathological and genetic overlap between patients^1^. Mutations in *Fused in sarcoma* (FUS) are found in 5% of ALS patients while pathological FUS aggregates are found in 10% of FTD patients^2–4^. The majority of FUS mutations reside within the C-terminus of the protein which contains the nuclear localising signal (NLS) and is essential for FUS to traffic between the nucleus and cytoplasm^5,6^. Under physiological conditions, FUS is a predominately nuclear protein which has a role in RNA processing^7^. However, a growing body of evidence has shown that FUS has an essential cytoplasmic role in neurons involved in transport and local protein translation, especially at the synapse. FUS has been shown to be located at both the pre- and post-synapse^8,9^ as well as interacting with GluA1, the AMPA receptor subunit, which is a post-synaptic protein^10^. Evidence points towards FUS being essential for dendritic reorganisation by regulating synaptic mRNAs^11,12^ with FUS depletion leading to a decrease in GluA1 expression resulting in supressed synaptic transmission and changes in synaptic maturation^10^. Notably, FUS has been implicated in the regulation of splicing and the transcription of synaptic mRNAs which could be involved in local translation at the synapse^13,14^. Mutations in the NLS lead to varying levels of accumulated mutant FUS in neuron terminals and have been shown to cause significant hypomethylation of arginine’s which decreases new protein synthesis^15^. Therefore, accumulating evidence points towards FUS having a vital cytoplasmic role for mRNA transport within axons and dendrites to facilitate local translation at the synapse^16^. However, how mutated cytoplasmic FUS contributes to synaptic degeneration still needs to be elucidated.

Mitochondria are essential for synaptic plasticity, spine development and general neuronal function with ATP demand being correlated to synaptic integrity within a dendrite^17^. The pathogenesis of numerous neurodegenerative diseases, including ALS, has been associated with mitochondrial dysfunction and demonstrates that mitochondrial function is essential for maintaining neuronal integrity^18–20^. In ALS specifically, there is accumulating evidence in both mouse and Drosophila models that degeneration of the NMJ, accompanied by mitochondrial abnormalities, is an early pre-symptomatic disease event^21–25^ FUS has been shown to interact with mitochondrial proteins (HSP60; ATP synthase β-subunit) and mutations in FUS which increase cytoplasmic FUS have been associated with mitochondrial fragmentation within neurons^26,27^. Additionally, it has been shown that a subset of FTD patients show an increase in FUS expression within damaged mitochondrial cristae^27^. It is therefore clear that healthy mitochondria are essential for synaptic functioning in the context of ALS, however the involvement of FUS is relatively unknown.

Here, we report in vitro and in vivo evidence that overexpression of mutant FUS causes differential synaptic defects which appear to depend on the level of mislocalised cytoplasmic FUS. Additionally, we have provided evidence for a relationship between synaptic and mitochondrial abnormalities due to mutant FUS by identifying a novel interaction between FUS and the mitochondrial anchor protein, syntaphilin (SNPH) which is essential for synaptic maintenance. These results indicate that FUS is intricately involved in synaptic and mitochondrial functioning and that the degree of mislocalised FUS can lead to specific abnormalities which contribute to neurodegeneration.

## Results

### FUS is enriched at, and colocalises with pre- and post-synapses in rat primary neurons

**Figure 1:**
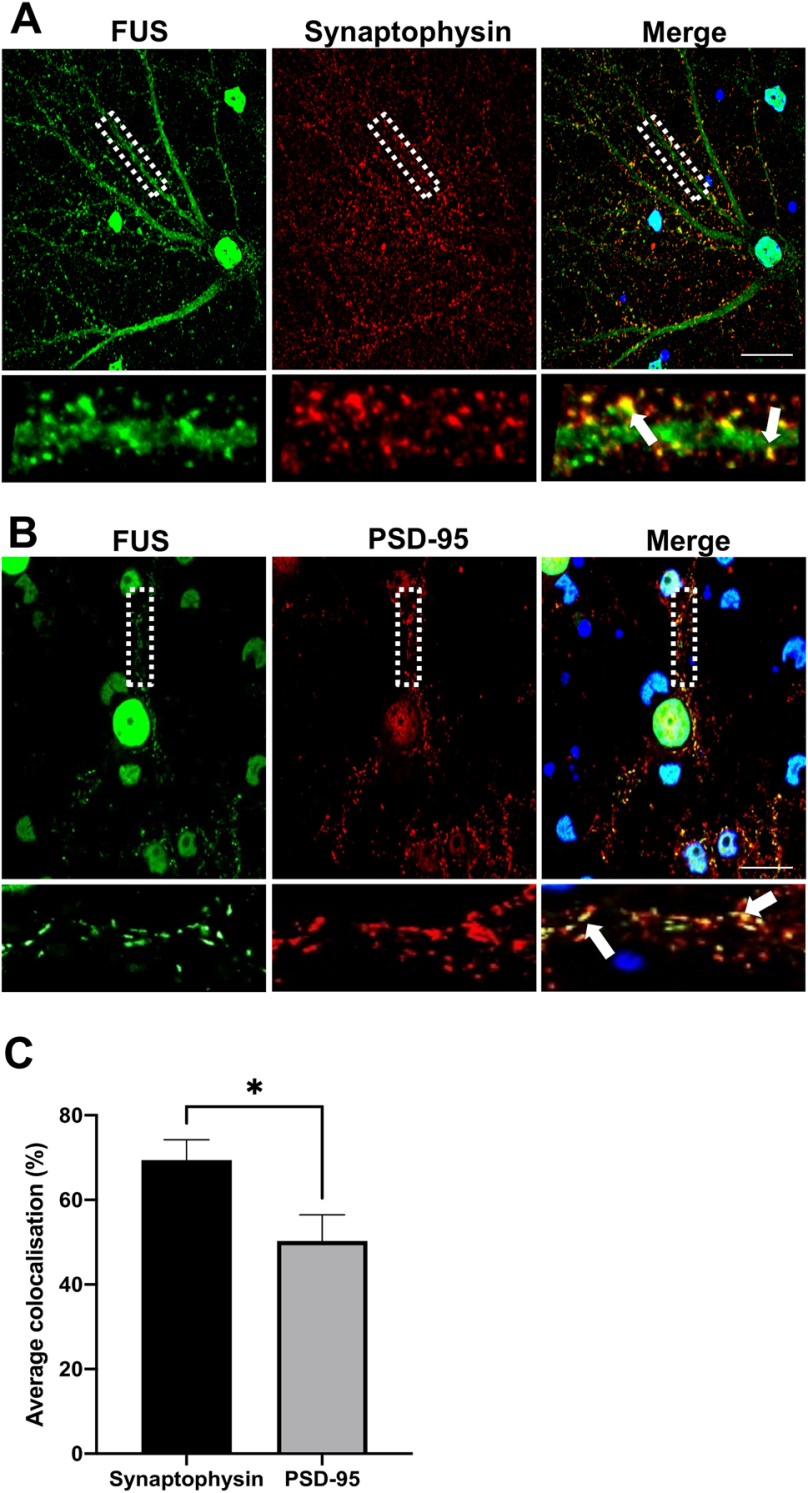
Subcellular localisation of FUS in primary cortical neurons. Immunofluorescent staining of DIV21 rat primary cortical neurons. (**A**) Representative confocal images of FUS (red) and presynaptic marker synaptophysin (SYN) (green) FUS was found to localise with synaptophysin puncta along neurites. (**B**) Representative confocal images of FUS (green) and the post synaptic marker PSD95 (Red). FUS was found to localise with PSD5 puncta along neurites. Selected regions of interest for A and B have been shown as magnifications as single and merged channels below the images of the respective neurons with white arrows indicating colocalisation. Nuclei are counterstained blue with DAPI. (**C**) Quantification of the subcellular localisation showed that FUS preferentially localised to the pre-synapse in these neurons (three neurites from nine different neurons from three independent experiments were analysed). *P < 0.05, unpaired students T-test. Scale bar = 20 μm.

To investigate whether FUS was localised to the synapse, we performed immunocytochemistry using rat primary cortical neurons to co-stain for endogenous FUS and pre- and post-synaptic markers. Neurons were aged to DIV21 to ensure expression of both pre- and post-synaptic markers. Besides its expected localisation within the nucleus, FUS was also localised within puncta in neurites (Fig. 1). FUS colocalised with 75% of synaptophysin puncta (Fig. 1A, SYN, pre-synaptic) and 50.25% of post synaptic density-95 (Fig. 1B, PSD-95) puncta. This indicates that FUS is present on both sides of synaptic buttons with a preferential localisation for the pre-synapse at DIV21 (p < 0.05).

### Mutant FUS leads to alterations at the synapse

**Figure 2:**
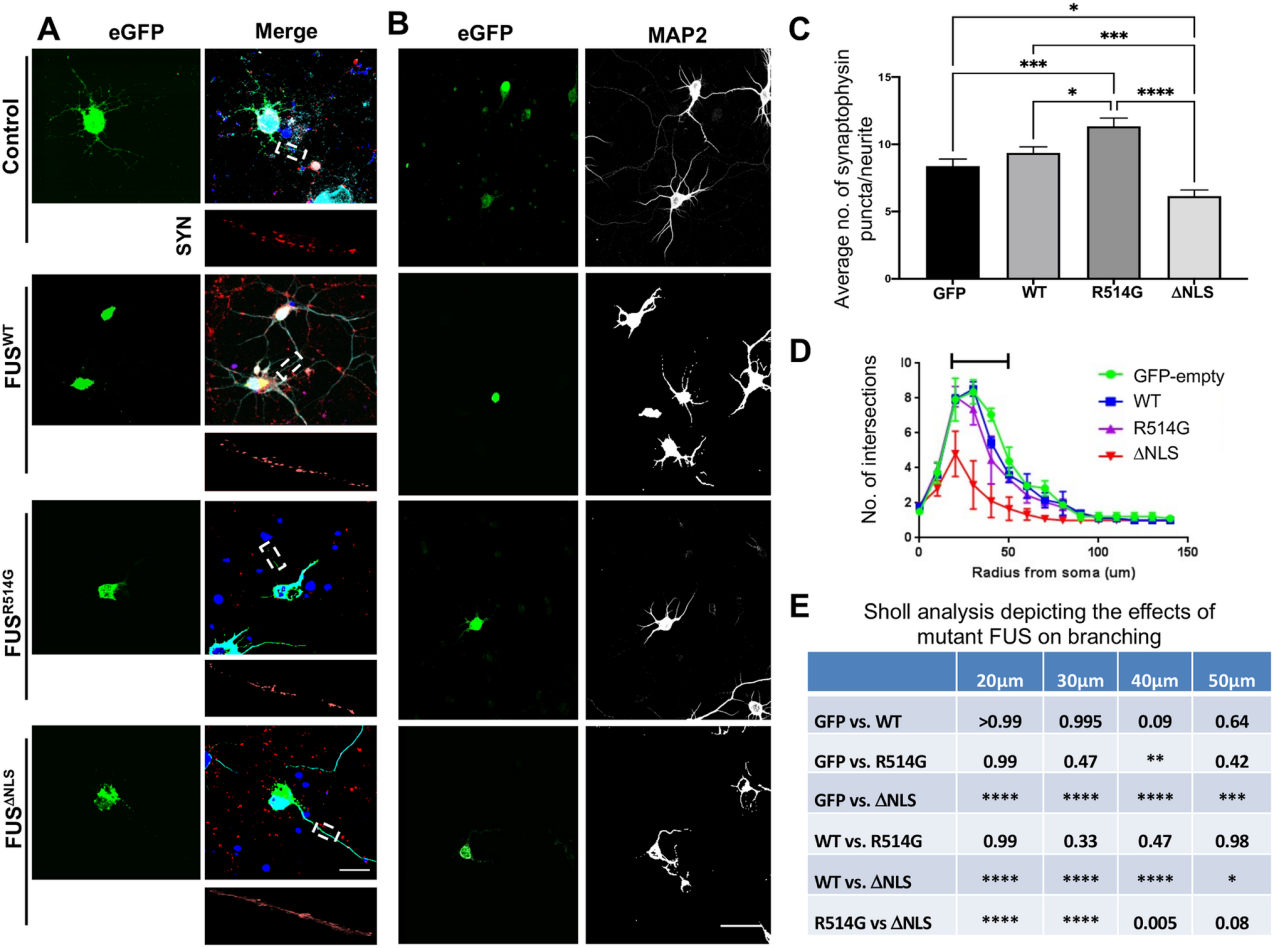
ALS-linked mutations in FUS lead to pre-synaptic alterations. (**A**) Representative confocal images of DIV8 rat primary cortical neurons transfected with eGFP, eGFP-FUS^WT^, eGFP-FUS^R514G^ or eGFP-FUS^ΔNLS^ (green) and stained for synaptophysin (red) and MAP2 (merge). Overexpressed mutant FUS leads to differing levels of cytoplasmic mislocalisation for both eGFP-FUS^R514G^ and eGFP-FUS^ΔNLS^. Smaller images below show a representative region of interest used for quantification. Nuclei were counterstained with DAPI. Scale bar = 10 μm. (**B**) Representative confocal images showing mutation specific changes to dendritic branching. Left panels show eGFP expression (green), right panels show MAP2 staining (greyscale). Scale bar = 100 μm. (**C**) Quantitative analysis comparing the number of pre-synaptic puncta between each mutation and control. There is a significant increase in the number of SYN puncta in the neurons transfected with eGFP-FUS^R514G^ compared to eGFP-FUS^WT^ (p < 0.05) and a significant decrease in puncta in neurites expressing eGFP-FUS^ΔNLS^ (p < 0.0001). Statistical analysis was performed using a One-Way ANOVA with a post-hoc Tukey’s multiple comparisons test; error bars are ± SEM (n = three neurites from five cells per condition, three independent replicates). Data represent mean synaptophysin puncta on each dendrite per 10 μm ± SEM. (**D**,**E**) Sholl analysis revealed a significant change in branching between 20–50 μm away from the soma with the eGFP-FUS compared to all other conditions (n = ten transfected cells from three individual replicates). Statistical analysis was performed using a two-Way ANOVA with a multiple comparisons test which compared the simple effects within each row; error bars are ± SEM. Significant results are represented by the black bar and presented in the table (**E**). *p < 0.05, **p < 0.01, ***p < 0.001 ****p < 0.0001.

To investigate whether mutations in FUS affected the synapse, we over-expressed two different mutant forms of FUS in primary neurons. R514G is an NLS point mutation originally identified by us in 2009 in a British Family and replicated in German ALS patients that results in a moderate increase of cytoplasmic FUS in cell culture models (FUS^R514G^)^2,6,28^. Secondly, we created a truncation mutation (K510X) that results in the loss of the entire NLS from the C-terminus of the protein showing a predominantly cytoplasmic localisation (FUS^ΔNLS^)^6^. These two contrasting mutations allow us to investigate mild and severe variants of FUS mutations to determine whether the degree of mislocalisation affects the observed phenotype. eGFP-FUS^WT^, eGFP-FUS^R514G^ and eGFP-FUS^ΔNLS^ were transfected into DIV6 rat primary neurons alongside control eGFP-only to investigate if a change in pre-synaptic SYN puncta was observed (Fig. 2). Quantification of the expression of the plasmids suggests all are expressed at the similar levels (Fig. S1). As expected eGFP-FUS^WT^ was predominantly nuclear whilst the mutant proteins misclocalised to the cytoplasm (Fig. 2A). There was no significant difference in the number of SYN puncta between the control and eGFP-FUS^WT^ conditions indicating that transfection of eGFP-FUS^WT^ did not influence the number of SYN puncta (Fig. 2C). However, there was a significant increase in the number of SYN puncta of the neurons in the eGFP-FUS^R514G^ compared to eGFP-FUS^WT^ (p < 0.05) and a significant decrease in the number of SYN puncta expressing eGFP-FUS^ΔNLS^ compared to eGFP-FUS^WT^ (p < 0.001) suggesting potentially different effects of the two mutations (Fig. 2C). Secondly, as mutant FUS has been shown to affect dendritic branching in mouse models, an analysis of this was undertaken by analysing the MAP2 staining of transfected neurons^16^. This showed that only transfection of eGFP-FUS^ΔNLS^ led to a significant decrease in the ability to grow dendritic branches compared to the control (Fig. 2B,D,E).

**Figure 3:**
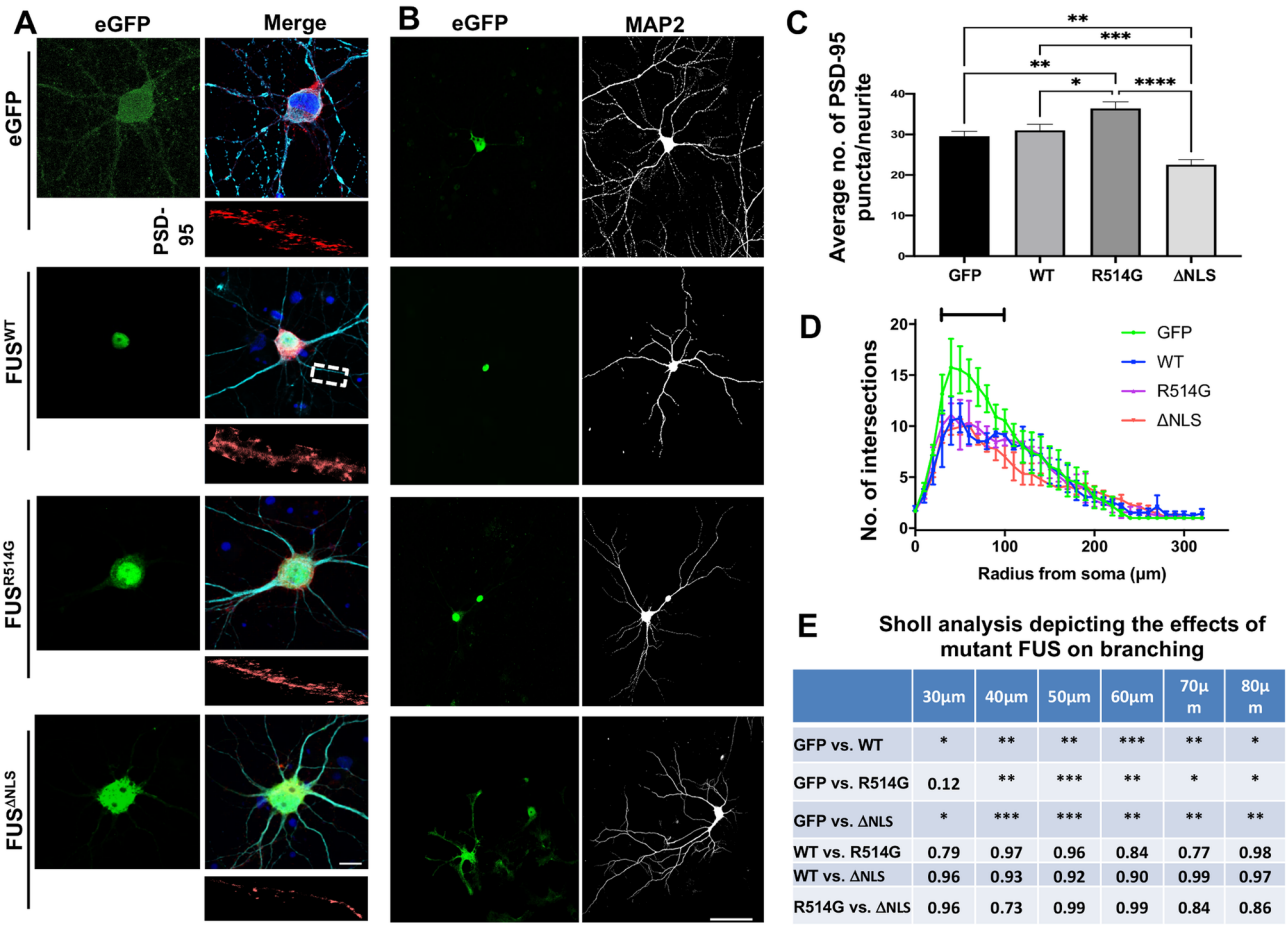
ALS-linked mutations in FUS lead to post synaptic alterations. Representative confocal images of DIV21 rat primary neurons transduced with eGFP, HA-FUS^WT^, HA-FUS^R514G^ and HA-FUS^ΔNLS^. Cells were stained for HA (green), PSD-95 (red) and MAP2 (merge). HA staining shows cytoplasmic mislocalisation of the mutant FUS. Regions of interest show magnified dendrites used to quantify the PSD-95. Nuclei were counterstained with DAPI. Scale bar = 10 μm. (**B**) Representative confocal images show HA-FUS expression (left) and dendritic branching shown by MAP2 (greyscale) staining (right). Scale bar = 100 μm. (**C**) Quantitative analysis comparing the average number of PSD-95 puncta per dendrite after transduction of WT and mutant FUS synaptic alterations. There was a significant increase in the number of PSD-95 puncta in the neurons expressing HA-FUS^R514G^ compared to HA-FUS^WT^ (P < 0.05) and a significant decrease in puncta for those cells expressing HA-FUS^ΔNLS^ compared to HA-FUS^WT^ (P < 0.001). Statistical analysis was performed using a One-Way ANOVA with a post-hoc Tukey’s multiple comparisons test; error bars are ± SEM (n = three neurites from five cells per condition, three independent replicates). Data represent mean PSD-95 puncta on each dendrite per 10 μm ± SEM. Scale bar = 100 μm. (**D**,**E**) Quantitative analysis of dendritic branching after transduction with WT and mutant FUS. Sholl analysis revealed no overall significant change when assessing how dendritic branching was affected by WT or mutant FUS (n = ten transfected cells from three individual replicates). Statistical analysis was performed using a two-Way ANOVA with a multiple comparisons test which compared the simple effects within each row; error bars are ± SEM. *p < 0.05, **p < 0.01, ***p < 0.001 ****p < 0.0001.

We also investigated if a similar effect would occur on the post-synaptic side by staining for the common post synaptic marker PSD-95. DIV14 Rat primary neurons were transduced with either HA-FUS^WT^, HA-FUS^R514G^ or HA-FUS^ΔNLS^ before being grown to DIV21 to ensure full development of the post synapse (Fig. 3A). As we observed with the pre-synaptic side, expression of HA-FUS^R514G^ resulted in a significant increase in PSD-95 puncta when compared to HA-FUS^WT^ (p < 0.05), whilst HA-FUS^ΔNLS^ led to a significant reduction in PSD-95 puncta compared to wildtype (p < 0.001) (Fig. 3A,C). In this case transduction of either WT or mutant FUS led to a reduction in branching compared to the control at this stage (Fig. 3B,D,E). This suggests that perhaps any increase in FUS, in this case by overexpression, may lead to synaptic disruption.

### Mutant FUS affects the NMJ in zebrafish

**Figure 4:**
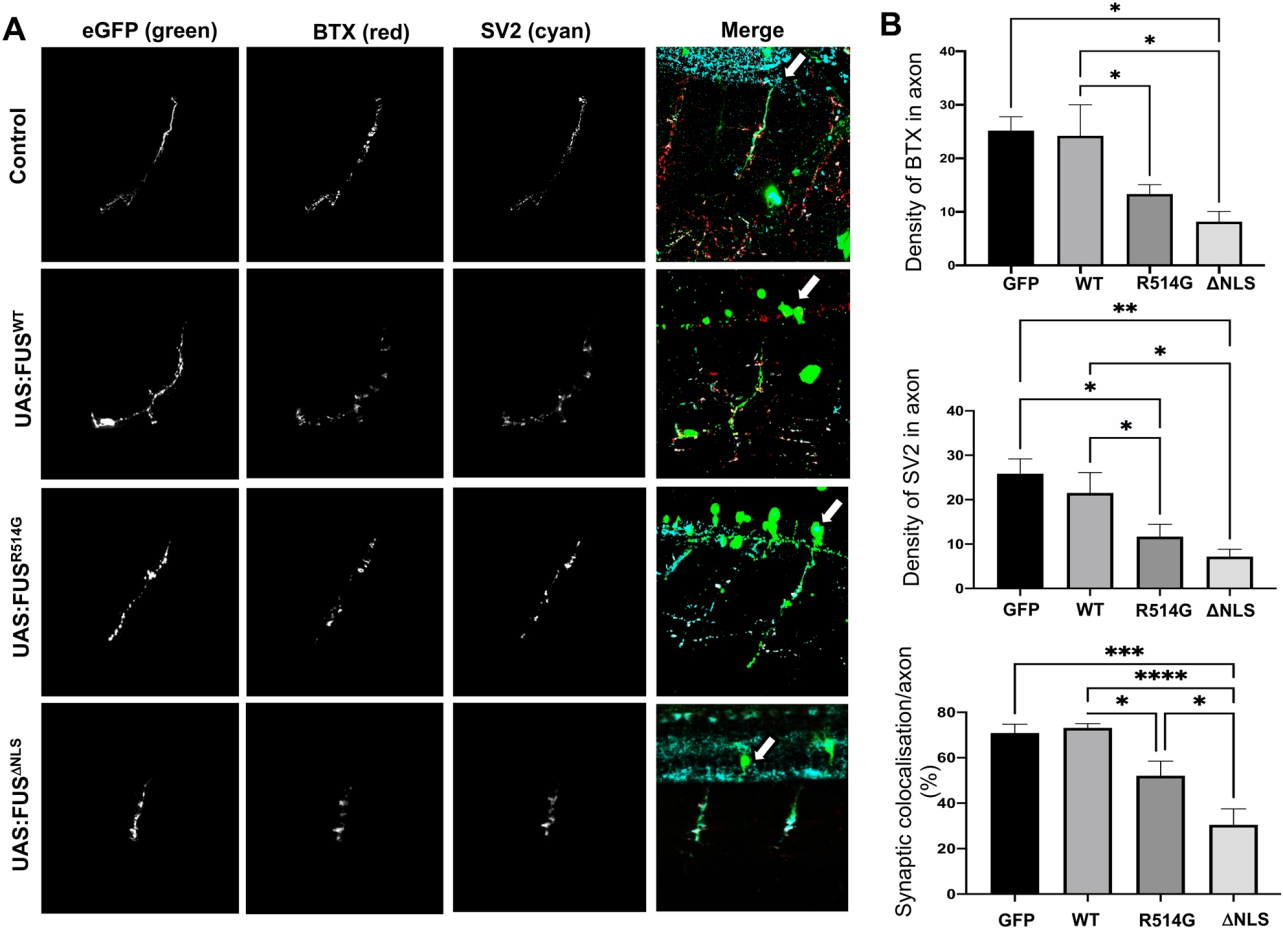
Zebrafish expressing mutant FUS show abnormal neuromuscular junctions and orphaned pre-synaptic endings. (**A**) Confocal images of long pec stage zebrafish trunk, lateral view, anterior to the right, imaged after microinjection with eGFP, eGFP-FUS^WT^, eGFP-FUS^R514G^ or eGFP-FUS^ΔNLS^. Merged images show the GFP-FUS (green) and SV2 (cyan) and BTX staining (red) which is indicated by a white arrow for each condition. Analysis was carried out on the greyscale images. Scale bar = 100 μm. (**B**) Quantitative analysis of the synaptic density of BTX, SV2 and synaptic colocalization. There was a significant reduction in the number of NMJs as shown by a reduction in both BTX (top) and SV2 (middle) staining in axons expressing eGFP-FUS^R514G^ (p < 0.05) and eGFP-FUS^ΔNLS^ (p < 0.05) compared to eGFP-FUS^WT^. The colocalisation between the SV2 and the BTX (bottom) was also reduced in axons expressing eGFP-FUS^R514G^ (p < 0.05) and eGFP-FUS^ΔNLS^ (p < 0.0001) compared to WT. Statistical analysis was performed using a One-Way ANOVA with a post-hoc Tukey’s multiple comparisons test; error bars are ± SEM. N = six different independent injections for each plasmid. *p < 0.05, **p < 0.01, ***p < 0.001 ****p < 0.0001.

To investigate whether synaptic changes were also seen in an in vivo model, we developed a transient transgenic approach in zebrafish to visualise and temporally monitor the motor neuron. In our model, expression of GFP-tagged FUS was driven by an mnx1 promoter which has previously been shown to drive expression in motor and interneurons of the zebrafish spinal cord^29,30^. This novel model results in expression of eGFP-FUS in a single motor neuron per injection and therefore we have used it to assess neuronal morphology and not behaviour. However, previous zebrafish models have shown a link between expression of mutant FUS and impaired locomotor activity due to synaptic changes^31,32^. We used the Gal4/UAS system to express eGFP-FUS^WT^, eGFP-FUS^R514G^ and eGFP-FUS^ΔNLS^ to explore changes specifically in ventrally innervating primary motor neurons. Previous data has shown that FUS is abundant at the NMJ and that denervation of the NMJ is an early pathological hallmark of ALS-FUS^22,33^. Therefore, we sought to establish whether mutant FUS disrupted the formation of the NMJ. Zebrafish co-injected with MNX1:Gal4 and either UAS: eGFP-FUS^WT^, UAS: eGFP-FUS^R514G^ or UAS: eGFP-FUS^ΔNLS^, were fixed and stained for the pre-synaptic NMJ marker SV2 and the post-synaptic NMJ marker alpha-bungarotoxin (αBTX) at 2 days post fertilisation (Fig. 4). We observed a significant reduction in the number of BTX and SV2 puncta in cells expressing eGFP-FUS^R514G^ (p < 0.05) and eGFP-FUS^ΔNLS^ (p < 0.05) compared to eGFP-FUS^WT^ (Fig. 4B). Furthermore, investigation into the degree of colocalization between BTX and SV2 showed a significant reduction for both eGFP-FUS^R514G^ (p < 0.05) and eGFP-FUS^ΔNLS^ (p < 0.0001) compared to eGFP-FUS^WT^ (Fig. 4B).

**Figure 5:**
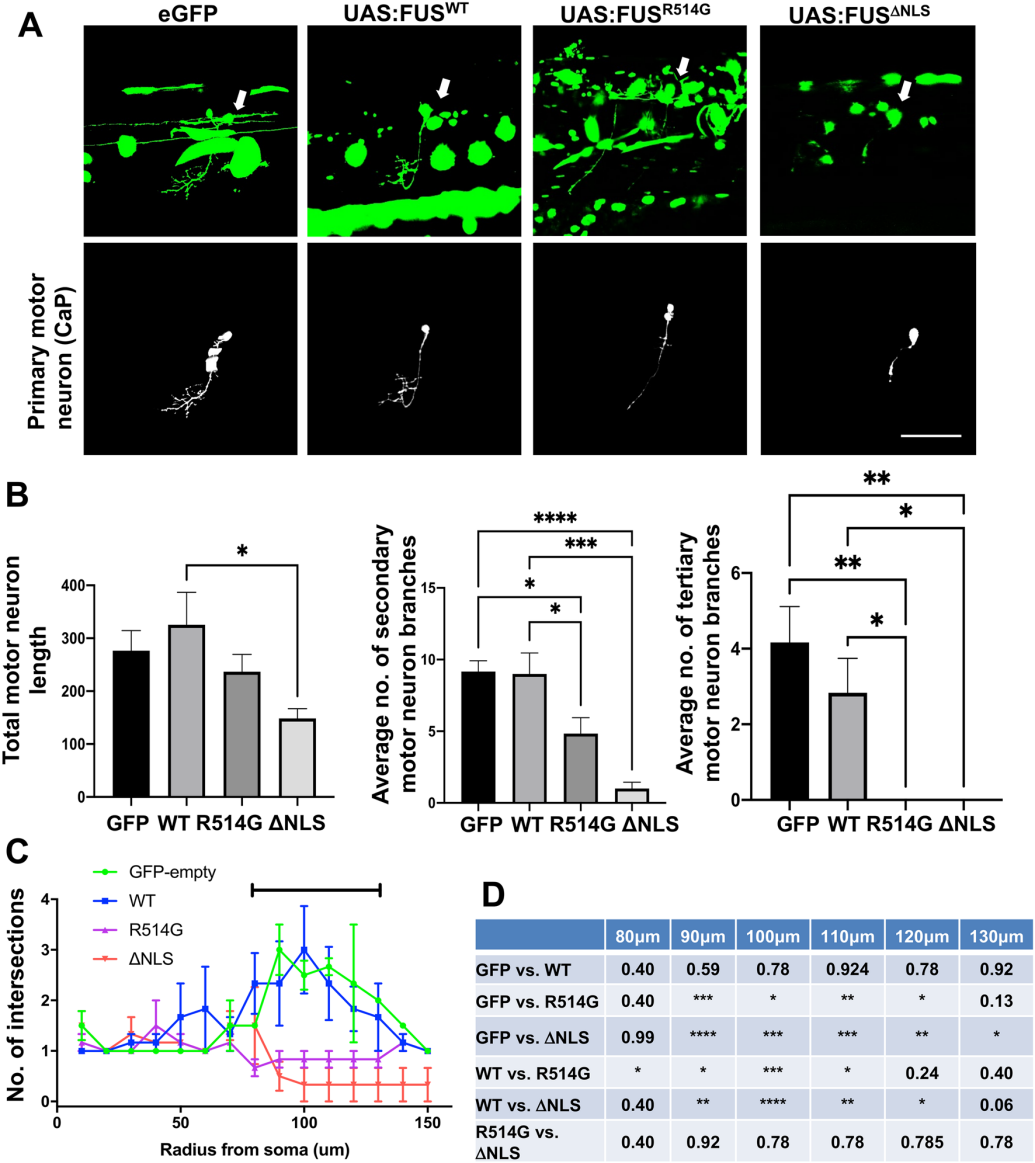
Mutant FUS expression in primary motor neurons affects axonal branching. (**A**) Confocal images of long pec stage zebrafish trunk microinjected with eGFP, eGFP-FUS^WT^, eGFP-FUS^R514G^ or eGFP-FUS^ΔNLS^. Images show the lateral view, anterior to the right. Top panels show an isolated GFP expressing motor neuron which are indicated by a white arrow (green) while bottom panels show a traced isolated motor neuron which was used to quantify axonal branching for each condition. Scale bar = 100 μm. (**B**) Quantitative analysis comparing total average axonal length where GFP-FUS is being expressed between each mutation and control injection (top left) shows a significant reduction in the eGFP-FUS^ΔNLS^ expressing length compare to eGFP-FUS^WT^. Data represent mean axonal length per 100 μm ± SEM. Analysis of the average number of secondary axonal branches (top right) showed a significant decrease in axons expressing eGFP-FUS^R514G^ (p < 0.05) and eGFP-FUS^ΔNLS^ (p < 0.001) compared to eGFP-FUS^WT^. Data represent mean number of secondary axonal branches per 100 μm ± SEM. When analysis was undertaken of the number of tertiary axonal branches (bottom), it was clear that whilst numbers were low, there were no tertiary branches in axons expressing eGFP-FUS^R514G^ or eGFP-FUS^ΔNLS^. Data represent mean number of tertiary axonal branches per 100 μm ± SEM was also compared between conditions. (**C**,**D**) Quantitative analysis of axonal branching after microinjection of eGFP-FUS^WT^ and mutant FUS in primary motor axons. Sholl analysis revealed a significant change between 80–130 μm away from the soma when assessing how axonal branching was affected by eGFP-FUS^WT^ or mutant FUS. Statistical analysis was performed using a two-Way ANOVA with a multiple comparisons test which compared the simple effects within each row; error bars are ± SEM. Significant results (represented by bar in **C**) are presented in the table (**D**). N = six different independent injections for each plasmid.

Following our discovery that the different mutations in FUS led to substantial synaptic defects, we sought to investigate the extent of the developmental defect of the misexpressing caudal primary motor neuron. Expression of MNX-specific eGFP-FUS^R514G^ or eGFP-FUS^ΔNLS^ resulted in a reduction in the length of the primary motor axon expressing eGFP-FUS when compared to eGFP-FUS^WT^ (Fig. 5A,B). Interestingly, this reduction only reached statistical significance in the ΔNLS mutant (p < 0.05, Fig. 5A,B). However, the expression of either mutants led to significant decreases in the number of secondary and tertiary branches that expressed eGFP-FUS. There was a significant reduction in secondary motor neuron branches in both the eGFP-FUS^R514G^ (p < 0.05) and eGFP-FUS^ΔNLS^ (p < 0.001) expressing zebrafish when compared to eGFP-FUS^WT^ (Fig. 5B). Whilst there are never large numbers of tertiary branches (eGFP control averaged only 4 tertiary branches), eGFP-FUS^WT^ motor neurons expressing cells presented an average of 2.83 tertiary branches per axon whilst none were detected in any analysed motor neurons expressing eGFP-FUS^R514G^ or eGFP-FUS^ΔNLS^ (Fig. 5A,B). This may indicate a delay in branching in neurons expressing higher levels of cytoplasmic FUS or as we are measuring the extent of the eGFP signal, rather than a neuronal marker in the motor neurons, this may reflect the extent to which mutant eGFP-FUS is transported in the motor axons. Subsequently, we sought to confirm this change in eGFP-FUS expressing axonal complexity in this in vivo system by using sholl analysis (Fig. 5C,D). This demonstrated that axonal branching was significantly reduced within primary motor neurons in both eGFP-FUS^R514G^ (p < 0.05–0.001) and eGFP-FUS^ΔNLS^ (p < 0.05–0.0001) expressing motor neurons compared to eGFP-FUS^WT^ (Fig. 5C,D).

### Mutant FUS affects neuronal mitochondria

**Figure 6:**
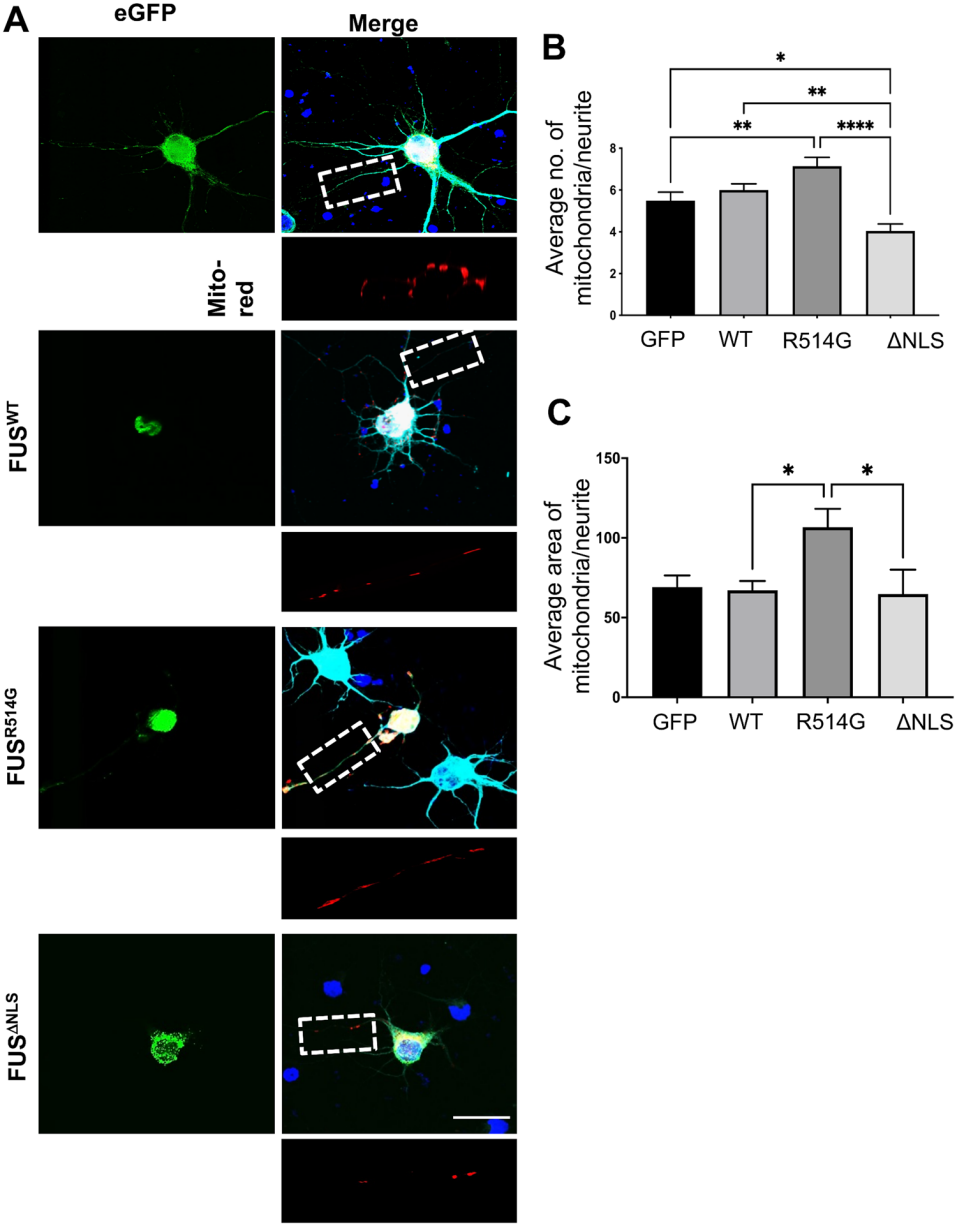
Expression of mutant FUS leads to mitochondrial abnormalities. (**A**) Representative confocal images of rat primary neurons co-transfected with eGFP, eGFP-FUS^WT^, eGFP-FUS^R514G^ or eGFP-FUS^ΔNLS^ (green) and Ds-MitoRed (red). Boxes show a region of interest that was used for quantification and magnified underneath. Scale bar = 10 μm. (**B**,**C**) Quantitative analysis of the average number and area of mitochondria within each dendritic branch after co-transfection with Ds-MitoRed and WT and mutant FUS. Statistical analysis was performed using a One-Way ANOVA with a post-hoc Tukey’s multiple comparisons test. n = three neurites from five different cells from three independent replicates were analysed for each co-transfection.

**Figure 7:**
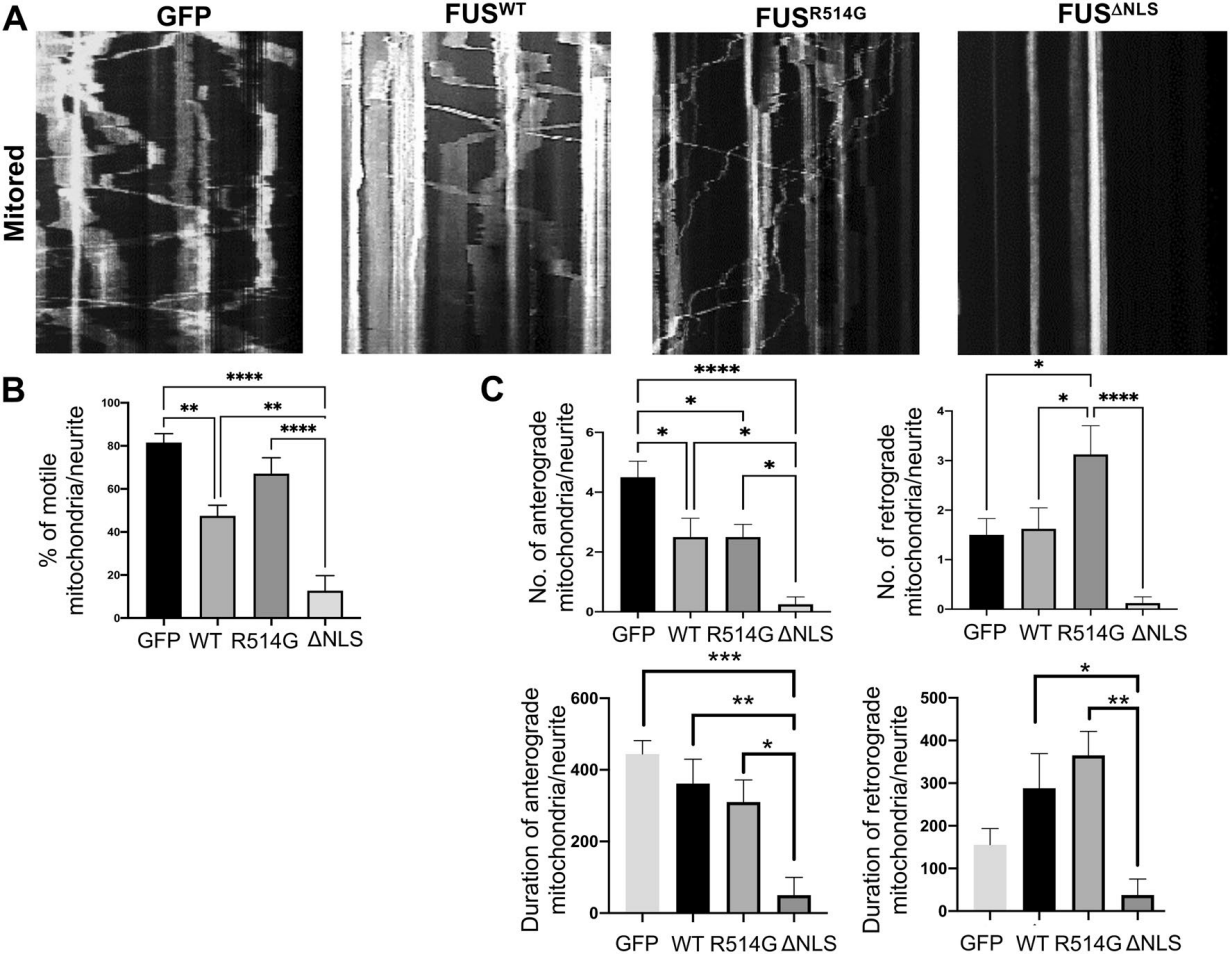
Mitochondrial motility is affected by mutations in FUS. (**A**) Kymographs showing mitochondrial movement in neurites over a 10 min period. The angle and number of lines indicates the speed, direction and number of mitochondria moving. N = eight neurites from three different independent replicates for each co-transfection. (**B**) Quantitative analysis of the kymographs showed significant changes to overall mitochondrial movement with eGFP-FUS^WT^ reducing the movement compared to the control (p < 0.01). eGFP-FUS^R514G^ showed an increase whilst there were very few mobile mitochondria in the eGFP-FUS^ΔNLS^ expressing neurons (p < 0.01). (**C**) Analysis of the number and duration of anterograde movement (left hand panels) shows no significant differences between the eGFP-FUS^WT^ and eGFP-FUS^R514G^ though there is a decrease in the average duration of the movement. In contrast there is a significant loss of movement and reduction in the in the eGFP-FUS^ΔNLS^ neurons compare to eGFP-FUS^WT^ (p < 0.05 number, p < 0.01 for duration). In contrast there is a significant increase in the retrograde number of mitochondria transported of eGFP-FUS^R514G^ (p < 0.05) and duration of movemement (p < 0.01) compared to eGFP-FUS^WT^ with almost a complete loss of movement in the eGFP-FUS^ΔNLS^ transfected cells. Statistical analysis was performed using a One-Way ANOVA with a post-hoc Tukey’s multiple comparisons test. *p < 0.05, **p < 0.01, ***p < 0.001 ****p < 0.0001.

We have previously shown that alterations to the morphology and a reduction in the number of mitochondria are very early disease events in a mouse model of ALS-FUS^22^. We proceeded to investigate if alterations to mitochondria could explain the observed in vitro and in vivo synaptic effects caused by mutant FUS. To do this we co-transfected rat primary neurons with eGFP, eGFP-FUS^WT^, eGFP-FUS^R514G^ or eGFP-FUS^ΔNLS^ together with DS-MitoRed, which localises to mitochondria and investigated the number and size of mitochondria within the neurites of individual neurons (Fig. 6). In the eGFP-FUS^R514G^ cells, there was a non-significant increase in the number of mitochondria compared to eGFP-FUS^WT^ expressing neurons (p = 0.12) whilst in eGFP-FUS^ΔNLS^ there was a significant loss of mitochondria compared to eGFP-FUS^WT^ (p < 0.01) (Fig. 6A,B). To investigate the health of the mitochondria we investigated morphological defects by assessing the size of the mitochondria themselves. Whilst there was no difference in size when comparing eGFP-FUS^ΔNLS^ to eGFP-FUS^WT^ neurons (p > 0.99), there was a striking increase in the average size of the mitochondria in cells transfected with eGFP-FUS^R514G^ compared to eGFP-FUS^WT^ (p < 0.05) (Fig. 6A,C). To investigate whether this affected mitochondrial transport, live imaging of the mitochondria in the transfected neurons was performed (Fig. 7A). An analysis of the overall motility of mitochondria in the transfected cells showed that there was a significant reduction in movement when eGFP-FUS^WT^ was transfected compared to the eGFP only control (Fig. 7B, p < 0.01). The presence of the eGFP-FUS^ΔNLS^ mutant led to an even greater reduction (Fig. 7B, p < 0.01) in the overall motility of mitochondria compared to any other condition. However, in contrast, there was a non-significant increase in movement in the eGFP-FUS^R514G^ transfected neurons (Fig. 7B, p = 0.34). To determine whether there were directional differences in the motility of the mitochondria, we analysed the anterograde or retrograde movement separately. Whilst both eGFP-FUS^WT^ and eGFP-FUS^R514G^ showed reduced anterograde movement compared to the eGFP control (Fig. 7C, p < 0.05), there was an even greater loss of movement in the eGFP-FUS^ΔNLS^ mutant (Fig. 7C, p < 0.0001). Furthermore, when assessing retrograde movement there was no difference when comparing eGFP-FUS^WT^ to the eGFP control (Fig. 7C, p = 0.66). There was however a significant increase when comparing eGFP-FUS^R514G^ to eGFP-FUS^WT^ or eGFP control conditions (Fig. 7C, p < 0.05). As with the anterograde movement, the presence of eGFP-FUS^ΔNLS^ resulted in almost no retrograde transport of mitochondria. Further we analysed the duration of the mitochondrial movement in each condition. In the anterograde direction that there is a slight reduction in the average length of mitochondrial movements when comparing eGFP-FUS^R514G^ to eGFP-FUS^WT^ (p = 0.9) and a significant decrease when comparing eGFP-FUS^ΔNLS^ to eGFP-FUS^WT^ (p = 0.002). In contrast, there is a slight increase in the average duration of each mitochondrial movement in the retrograde direction when comparing eGFP-FUS^R514G^ to eGFP-FUS^WT^ (p = 0.77) which matches the increased motility seen. As expected, there is a significant decrease in the duration of mitochondrial movement when comparing eGFP-FUS^ΔNLS^ to eGFP-FUS^WT^ (p = 0.01) which fits with the almost stationary mitochondria seen in this condition. Interestingly our results demonstrate a striking mitochondrial phenotype for each respective mutation as FUS^ΔNLS^ leads to a reduction in mitochondria and a complete loss of mitochondrial movement whereas FUS^R514G^ appears to lead to more swollen mitochondria which are more frequently moving towards the soma.

### FUS localises with the mitochondrial anchor, syntaphilin (SNPH)

**Figure 8:**
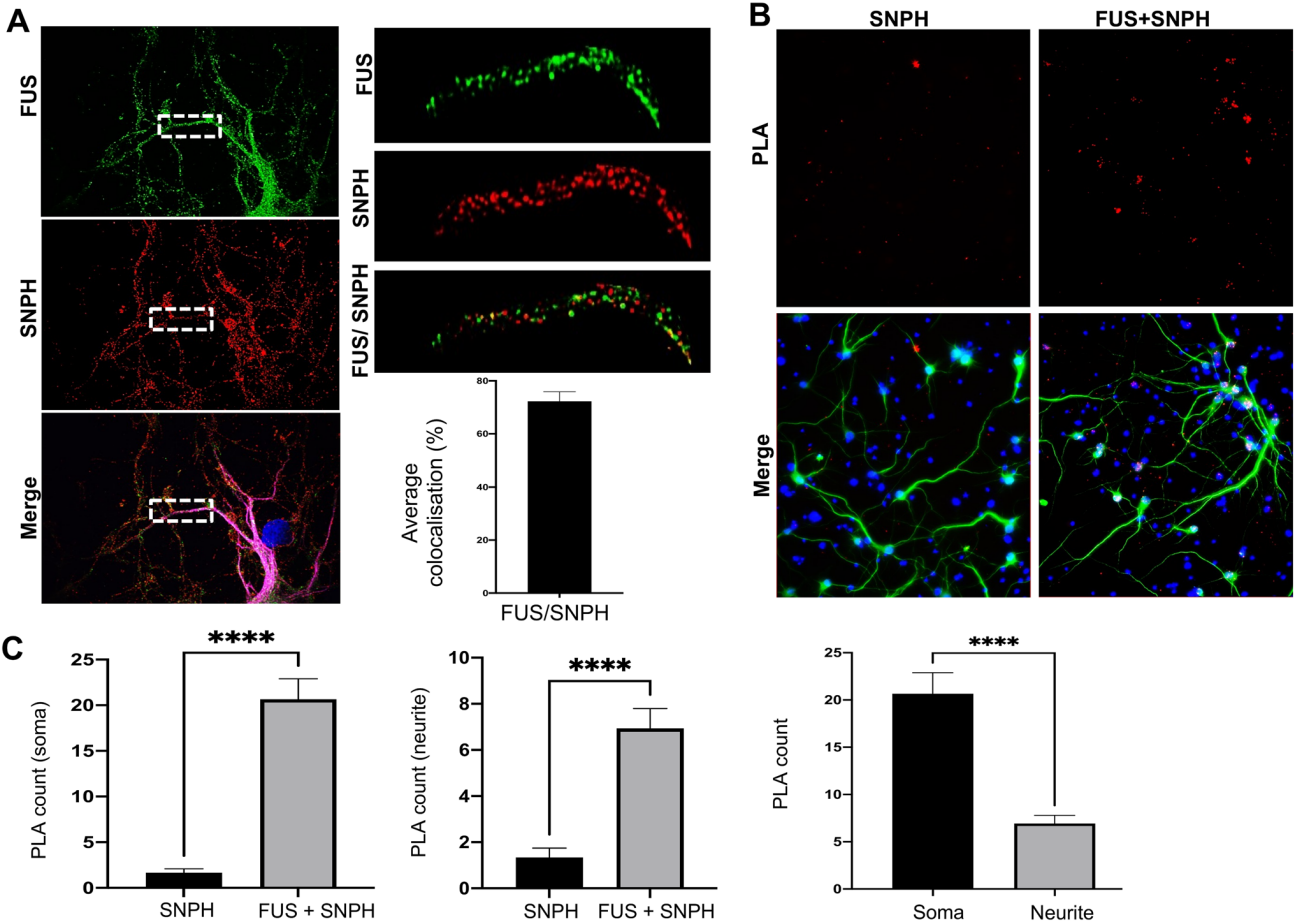
FUS Interacts with the mitochondrial anchor protein, Syntaphillin. (**A**) Representative super-resolution images of rat primary cortical neurons stained for endogenous FUS (green), Syntaphillin (red) and MAP2 (merge). Quantification of the subcellular localisation showed that FUS and SNPH preferentially localised within neurites (N = three dendrites from ten different neurons). Scale bar = 20 μm. Statistical analysis was performed using a paired student’s T-test; error bars are ± SEM. (**B**) Representative images of PLA in rat primary neurons. PLA (red) was used to assess interactions within soma and neurites which were determined through MAP2 staining (green). Nuclei were counterstained with DAPI. Scale bar = 100 μm (**C**) Quantification of PLA interactions within soma and neurite for both SNPH. There was an interaction between FUS-SNPH shown in the soma and neurites whilst there appears to be a stronger interaction with FUS-SNPH in the soma when compared to neurites. N = five cells and three neurites per cell were analysed from three different independent replicates. ****p < 0.0001.

We next examined if FUS interacted with mitochondrial proteins directly which could explain these effects of mutant FUS. We specifically looked at Syntaphilin (SNPH) due to its significant role in mitochondrial anchoring and its relationship to the synapse^34,35^. In order to confirm that SNPH was localised to mitochondria, we used super resolution microscopy (iSIM) to investigate the co-localisation of endogenous SNPH and a mitochondrial marker, TOM-20. This showed that TOM20 puncta colocalised with ~ 80% of SNPH (Fig. S2). Next, we investigated the localisation of endogenous FUS and SNPH within neurons. Results indicate that both FUS and SNPH form puncta in the soma and neurite and that FUS puncta colocalised with ~ 72% of SNPH (Fig. 8A). In order to determine whether FUS and SNPH more closely interacted, we used a Proximity Ligation Assay (PLA) which detects protein–protein interactions which are < 40 nm apart. PLA indicated that FUS was in close proximity to SNPH (Fig. 8B,C) and that a stronger localisation was found in the soma when compared to neurites (Fig. 8B,C, p < 0.0001).

### Mutant FUS leads to changes in interactions with SNPH

**Figure 9:**
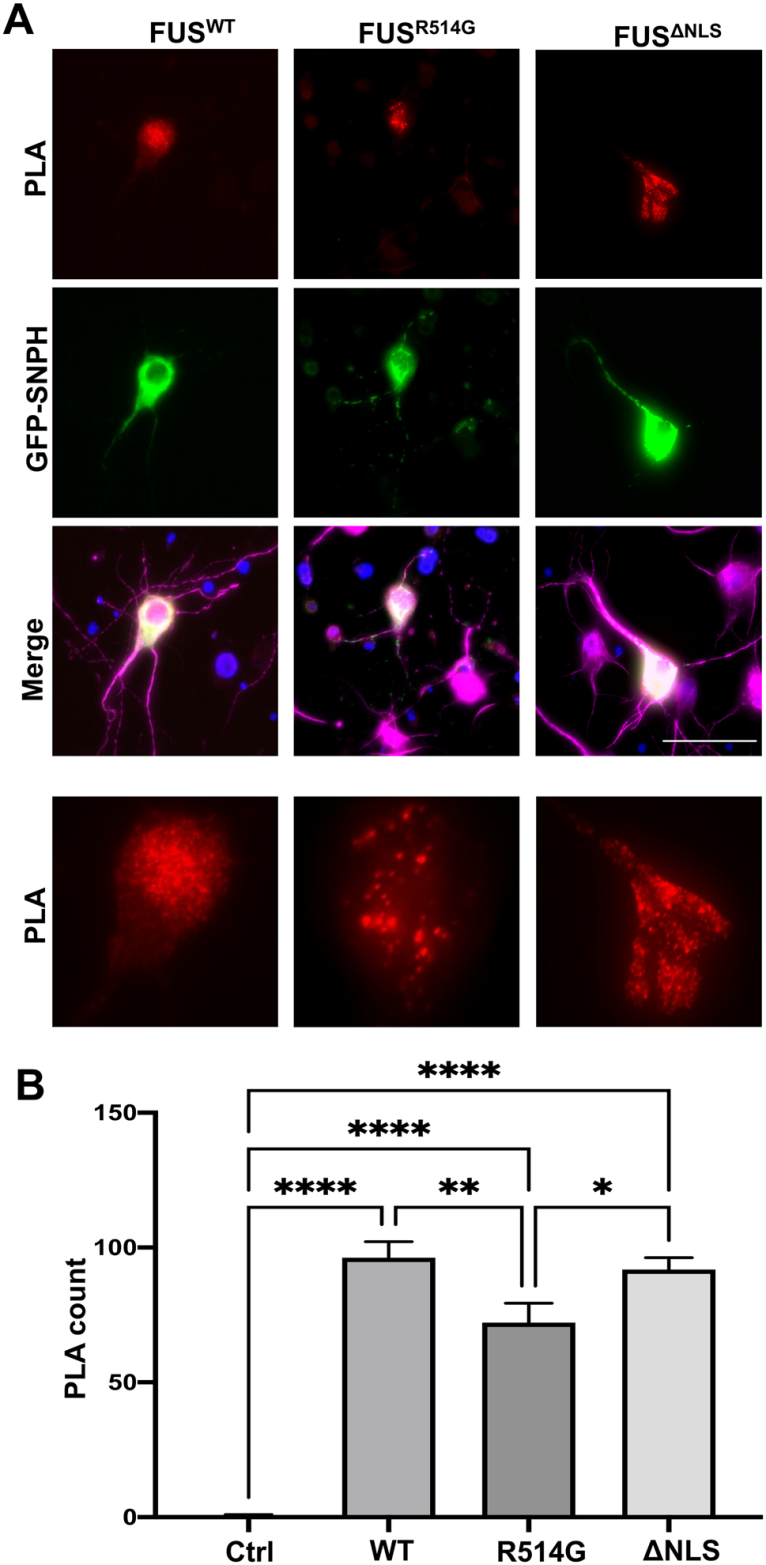
Overexpression of FUS mutations and GFP-SNPH alters FUS-SNPH interactions in transfected neurons. (**A**) Representative images of primary cortical neurons transfected with HA-FUS^WT^, HA-FUS^R514G^ or HA-FUS^ΔNLS^ (green). eGFP-SNPH is shown for each transfected cell which allowed PLA (red) between HA and eGFP to assess interactions within the cell body for each neuron analysed. Nuclei were counterstained with DAPI. A zoomed in image of the soma for each condition has been provided to show the change in FUS-SNPH interactions, Scale bar = 50 μm (**B**) Quantification of PLA interactions within soma for HA and eGFP. There is a significant decrease in the FUS-SNPH interaction in HA-FUS^R514G^ transfected cells compared to HA-FUS^WT^ (p < 0.01) and a non-significant decrease in the FUS-SNPH interaction in the HA-FUS ^ΔNLS^ compared to HA-FUS^WT^ (p = 0.6277). Statistical analysis was performed using a One-Way ANOVA with a post-hoc Tukey’s multiple comparisons test; error bars are ± SEM. N = five cells were analysed from three different independent replicates. Single antibody controls were performed for the antibody which shows a mainly diffuse pattern. *p < 0.05, **p < 0.01, ***p < 0.001 ****p < 0.0001.

Having shown that FUS co-localises with SNPH, we investigated whether overexpression of WT and mutant FUS would change the FUS-SNPH localisation pattern as mutations in FUS are known to alter interactions with mitochondrial proteins such as HSP60 and ATP5B^25,26^. HA-FUS^WT^, HA-FUS^R514G^ and HA-FUS^ΔNLS^ were transfected into rat primary neurons, along with eGFP-SNPH to ensure there was sufficient signal from both proteins, and a PLA was performed (Fig. 9 and Fig. S3). PLA indicated that there was a significant co-localisation between HA-FUS^WT^ and eGFP-SNPH and this was predominantly focused in the soma. The presence of HA-FUS^R514G^ led to a significant decrease in FUS-SNPH co-localisation when compared to HA- FUS^WT^ (Fig. 9, p < 0.01) which correlates with the increased movement of mitochondria seen with the R514G mutant. Surprisingly though there was no alteration of HA-FUS^ΔNLS^ compared to HA- FUS^WT^ suggesting that although this mutation almost abolishes the movement of mitochondria, it may not be due to an alteration in its potential interaction with SNPH as measured here.

### Protein translation is impaired in the presence of mutant FUS

**Figure 10:**
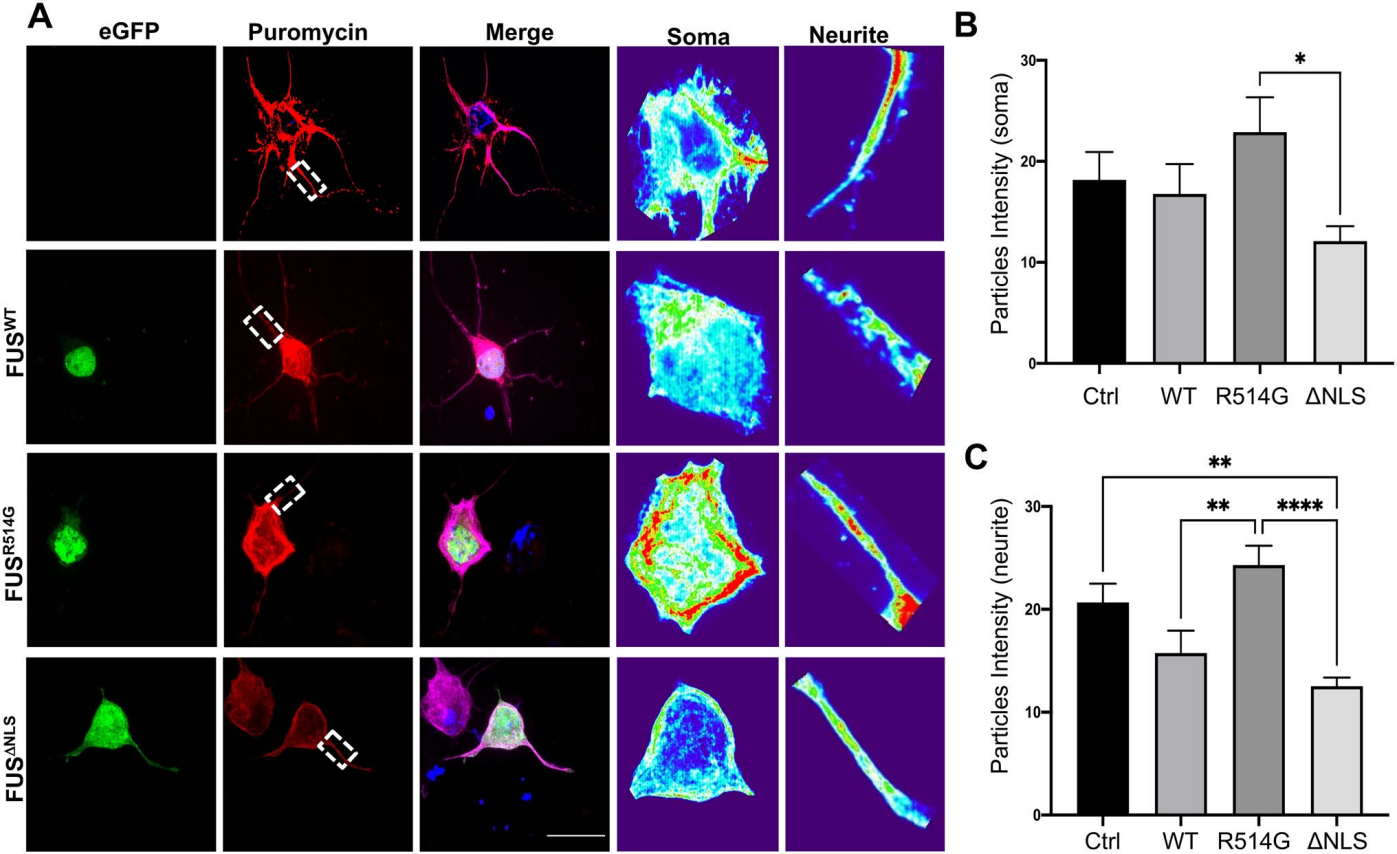
Overexpression of FUS mutations alters protein translation within transfected neurons. (**A**) Representative images of primary cortical neurons transfected with eGFP-FUS^WT^, eGFP-FUS^R514G^ or eGFP-FUS^ΔNLS^ (green) and stained for puromycin (red) and MAP2 (merge). Zoomed in images of each soma and neurite which were analysed has been included for each condition and presented in a heatmap to show changes more clearly. (**B**) Quantification of average intensity of puromycin puncta within soma show a non-significant decrease when eGFP-FUS^ΔNLS^ is compared to eGFP-FUS^WT^ (p = 0.5704). Comparison of eGFP-FUS^WT^ to control showed no significance (p = 0.2041) whereas comparison of eGFP-FUS^WT^ to eGFP-FUS^R514G^ showed a non-significant increase (p = 0.4042). However, comparison of eGFP-FUS^R514G^ to eGFP-FUS^ΔNLS^ showed significant decrease (p < 0.05). (**C**) Quantification of protein translation in neurites showed a significant increase when eGFP-FUS^ΔNLS^ or eGFP-FUS^wt^ was compared to eGFP-FUS^R514G^ (P < 0.0001 and p < 0.01 respectively). Furthermore, a significant decrease was observed when control was compared to both eGFP-FUS^ΔNLS^ (P < 0.01) while a non-significant decrease was seen for comparison against and eGFP-FUS^wt^ (p = 0.2041). When eGFP-FUS^ΔNLS^ was compared to eGFP-FUS^wt^, no significant change was observed (P = 0.5704). Statistical analysis was performed using a One-Way ANOVA with a post-hoc Tukey’s multiple comparisons test; error bars are ± SEM. N = five cells were analysed from three different independent replicates. *p < 0.05, **p < 0.01, ***p < 0.001 ****p < 0.0001.

Previously, it has been shown that mutations in FUS reduce axonal protein synthesis^36^ and so to determine whether we saw a similar phenotype, we investigated whether protein translation was affected in our cellular model. We used the surface sensing of translation (SUnSET) assay in which puromycin, a structural analogue of aminoacyl tRNAs, is incorporated into nascent polypeptides and prevents elongation, allowing us to directly monitor translation^37^. Primary cortical neurons were transfected with eGFP-FUS^WT^, eGFP-FUS^R514G^ and eGFP-FUS^ΔNLS^ respectively before puromycin treatment and analysed by measuring the intensity within the soma and neurites (Fig. 10A). The presence of eGFP-FUS^ΔNLS^ led to a small non-significant decrease in protein translation in the soma when compared to eGFP-FUS^WT^ (Fig. 10B, p = 0.5704). In contrast, eGFP-FUS^R514G^ led to a small increase in translation in the soma when compared to eGFP-FUS^WT^ (Fig. 10B, p = 0.4042). Analysis of the protein synthesis in the neurites showed that there was a similar pattern that this time reached significance in the eGFP-FUS^R514G^ transfected neurons when compared to eGFP-FUS^WT^ (Fig. 10C, p < 0.01) whilst eGFP-FUS^ΔNLS^ still showed a small decrease when compared to eGFP-FUS^WT^ (Fig. 10C, p = 0.5704). Interestingly, overexpression of eGPF-FUS^WT^ alone reduced the amount of translation compared to the control which matches previous data regarding mitochondrial movement suggesting that the two processes are closely linked.

## Discussion

In this study, we have shown that mutations in FUS led to alterations in synaptic protein expression and reduced the complexity of neurites and axons in vitro and in vivo and that these defects correspond to mitochondrial abnormalities observed in neurites with each respective FUS mutant. More importantly we have generated novel data showing FUS co-localises with the mitochondrial anchor protein SNPH in neurons, and that mutations in FUS alter this. Finally, we show that mutant FUS alters protein translation at the soma and in particular, neurites. Overall, we have presented evidence which supports a possible relationship between synaptic and mitochondrial function and neuronal health in which FUS appears to be a key player. It is well known that neuronal mitochondria are highly dynamic and transported through the neuron to regions of high metabolic demand. Therefore, it is possible that ATP demand is correlated to synaptic integrity within a neuron^17^ and an increase in mitochondrial number in FUS^R514G^ could explain the observed increase in synapses. Conversely, a decrease in mitochondrial number and therefore ATP, could explain the associated decrease in synapses in FUS^ΔNLS^ leading to the degeneration of the neuron. Further, there is evidence in primary cortical neurons that alterations in mitochondrial transport affect synaptic activity indicating that there is a link between mitochondrial and synaptic changes^38^. Taken together, this data adds to the accumulating body of evidence that FUS plays a role in mitochondrial and synaptic function, and that the level of mislocalised cytoplasmic FUS can lead to varying effects on neuronal function^8,26^. Future studies into whether mitochondrial physiology is affected by mutations in FUS will help us to understand the relationship between increased cytoplasmic FUS, mitochondrial health and trafficking, and synaptic activity.

One of the more interesting aspects of this work was that specific FUS mutations can lead to different cellular phenotypes in vitro*,* suggesting that the degree to which FUS is mislocalised can have differential downstream consequences. Even an increase in the amount of wildtype protein was sufficient to cause some cellular phenotypes such as reducing mitochondrial movement. Given that there are ALS patients who have 3’ UTR mutations^39^ that lead to an increase in wildtype protein and that there are mouse models in which this increase alone is sufficient to cause an ALS like phenotype, it is not a surprise that we see such occurrences. Moreover, when we introduced a mutation that specifically and moderately increases cytoplasmic FUS (FUS^R514G^), this results in an increase in synapses, and mitochondrial number, size, and speed of axonal transport. Whereas FUS^ΔNLS^, a mutation that results in complete abolishment of the nuclear-localising signal and a very large increase in cytoplasmic FUS, contrasts FUS^R514G^ by demonstrating synaptic and mitochondrial deficit with a complete loss of axonal transport. Patients with truncation mutations suffer from a very young onset and aggressive form of ALS^28^. In comparison those with a FUS^R514G^ mutation have a later onset and longer form of the disease though this is still often more severe than those with the sporadic form of the disease (2; 5). This suggests that the FUS^R514G^ phenotype might represent an early disease response to the increased cytoplasmic FUS and that the FUS^ΔNLS^ phenotype might mimic an aggressive end stage timepoint. It is worth noting that we see different but internally consistent phenotypes in vivo and in vitro for FUS^R514G^. This may be due to the increased sensitivity of the zebrafish motor neurons to mislocalised cytoplasmic FUS, compared to somewhat more resilient cortical neurons, as assessed in our cell culture studies. This may reflect why motor neurons are selectively vulnerable in disease.

In this study, we showed that each mutation causes significant changes in mitochondrial transport. Mitochondria are transported anterogradely from the soma towards the synapse due to the high metabolic demand^34^. We show that FUS and SNPH localise together in neurites. This is of importance as SNPH acts as a stable anchor for mitochondria and is essential for synaptic modification and functionality by ensuring the presence of mitochondria near synapses^34^. Interestingly, neurons overexpressing FUS^R514G^ show a decrease in co-localisation with SNPH within the soma. This observation fits with our data, showing a greater number of motile mitochondria being transported within the neuron in cells transfected with FUS^R514G^. This altered interaction could explain both the potential increase in mitochondria numbers in the neurite and the increased retrograde transport we observe within our in vitro dataset. It is possible that the neuron is trying to compensate for the excess cytoplasmic FUS and stay functional by taking damaged mitochondria back to the cell body to be degraded^40^. However, we observed a non-significant decrease in FUS-SNPH co-localisation in neurons expressing FUS^ΔNLS^ within the soma and neurite. This suggests that although there is a similar co-localisation pattern when compared to FUS^WT^, we also showed that there were fewer mitochondria anyway so those that exist could be trapped within the soma or stationary in the neurite and not being transported. This could explain the decrease in mitochondrial transport and the overall number of mitochondria. It is also interesting to note that FUS and another ALS protein, ANXA11, are also found in G3BP1 stress granules linking them to similar cellular functions^41,42^ In addition, recent work has shown that ANXA11, acts as a link between lysosomes and hnRNP-containing RNA granules for axonal transport^41^ and it may be that FUS acts as link between RNA and mitochondria in a similar manner.

Our data also confirms previous data showing that mutations in FUS affect protein synthesis^36,43^. Moreover, translation defects appear to be specific to the degree of mislocalised cytoplasmic FUS depending on the mutation present. As with our previous data, FUS^R514G^ led to an increase in translation in affected soma and neurites whereas FUS^ΔNLS^ led to a decrease. Previous reports have demonstrated that mutant FUS interacts with polyribosomes and that a toxic ‘gain of function’ in the cytoplasm affects translation^43^. Therefore, it is likely that the changes in global translation alongside the mitochondrial abnormalities we observe lead to the synaptic abnormalities exhibited by each specific mutant. It has previously been shown that mitochondria act as a local fuel source and that disruption of these mitochondria affects local translation^44^. Further mutations in the endosome protein Rab7a, which cause Charcot-Marie-Tooth Disease type 2B, result in altered axonal protein synthesis and trafficking of mitochondria^45^. This suggests that there are strong links between mitochondrial transport, endosomes and local translation.

We have presented in vivo and in vitro evidence that FUS is essential for maintenance of neuronal health and that specific FUS mutations can cause differing mitochondrial and synaptic disruption, depending on the degree of cytoplasmic mislocalisation. This might explain some previously conflicting reports on the effect of mutations in FUS on neuronal function. Future studies will be needed to better understand the potential interaction between FUS-SNPH to prove if changes in the co-localisation could partially explain the synaptic and mitochondrial defects observed in vitro and in vivo seen here.

## Methods

### Cell culture

All neuronal culture techniques were performed under sterile conditions. Coverslips were coated with 1% Poly-D-lysine hydrobromide in PBS (Sigma) and incubated at 37 °C overnight and washed with PBS prior to the seeding of rat primary cortical neurons at 70,000 cells/ml in 500 μl/well.

### Animal experiments

All animal experiments have been authorised by the KCL ethics Review Committee and under the HO license 70/7577. All animal experiments were performed in accordance with the relevant guidelines and following regulated procedures and all authors complied with the ARRIVE guidelines for animal research. The study is reported in accordance with the ARRIVE guidelines and we have presented all details that allow for accurate follow up including group sizes, age, and detailed experimental procedures for each animal experiment.

### DNA transfection

For each transfection, 500 ng DNA was mixed with 1 μl of Lipofectamine 2000 (Invitrogen) in 25 μl of HEPES (Gibco) and DMEM (Gibco) solution per well. Prior to addition of DNA, coverslips were removed and placed in a 350 μl of fresh media (without pen/strep). DNA mix was incubated for 1 h at 37 °C before adding in a drop wise manner to DIV6 neurons. 6–8 h post transfection coverslips were replaced in the old media, before fixation 48 h later.

### Viral transduction

Virus was added to rat primary neurons at DIV14 to achieve an infection rate of 1 × 107 virus particles/ml. Following the day of transduction, 250 μl of cortical media was removed and 300 μl of fresh cortical media was added onto the cells before fixation occurred at DIV21.

### Immunofluorescence

Primary neurons were fixed in 4% paraformaldehyde for 10 min and then ice cold methanol for 10 min before being probed with selected primary antibodies overnight at 4 °C. Antibodies were used as follows rabbit anti-synaptophysin I (1:300, Synaptic Systems), rabbit anti-FUS (1:300, Sigma), mouse anti-FUS (1:200, Proteintech), mouse anti-PSD95 (1:500, AbCam), mouse anti-HA (1:1000, CoVance), chicken anti-MAP2 (1:1000, AbCam), mouse anti-FUS (H6) (1:200, Santa Cruz), rabbit anti-SNPH (1:200, ProteinTech). Goat anti-rabbit, anti-mouse or anti-chicken Alexaflour 488/564/640 secondary antibodies (1:500, Invitrogen) were added the following day for two hours at room temperature in the dark. DAPI (4ʹ,6-diamidino-2-phenylindole (Sigma) counterstaining (1.25 μg/ml) was added as a nuclear stain before cover slips were mounted.

### Quantitative image analysis

Images were taken on a Leica TCS-SP5 laser scanning confocal microscope and imaged at × 63 with a numerical aperture of 1.4 (with a digital zoom of 2.5). Images were taken at 10–14 Z-sacks with 0.5 μm increment before being processed in Image J.

### Colocalisation analysis

Images of selected neurites were extracted, and their length (100 μm) was recorded. After splitting each channel, a Gaussian and median filter (with a radius of 10 pixels) was applied to the channel of interest, individually. The median image was subtracted from the Gaussian channel and a threshold was selected. Particles were then analysed from the channel before overlaying puncta onto the other channel of interest to allow measurement of colocalization which was determined by subtracting the area of the overlaid puncta from the under laid puncta (colocalisation was only counted if 50% and over). Analysis was performed on three separate dendrites per cell (N = 9) from three individual experiments at DIV21. All statistical analysis to determine significance between groups was performed using GraphPad Prism 9 using a student’s T-Test.

### Analysis of synaptic puncta and mitochondria

Maximum projection images were converted to 8-bit grayscale and individual channels were then used to select the threshold which was kept consistent to the control (eGFP) channel. Puncta and mitochondria were thresholded to be bigger than five pixels in size. Images were obtained from three independent experiments and three dendrites from five different cells were analysed for each repeat for each condition. All statistical analysis to determine significance between groups was performed using GraphPad Prism 9 using a One-Way ANOVA with post-hoc tukey’s multiple comparisons test.

### Analysis of dendritic complexity

Maximum projection images of the MAP2 channel were then converted into an 8-bit gray scale image and dendrites were traced using an available plugin (Neuron J). After tracing, sholl analysis was performed to assess dendritic complexity from the soma at 10 μm increments. Sholl analysis is a quantitative measure of the shape and/size of a dendritic tree. To measure dendritic complexity, concentric circles were drawn from the centre of the neuron and the number of times each dendritic branch intersects, branching is assessed (number of intersections divided by area against distance). Traces were analysed from 10 individual cells across three individual experiments per condition. All statistical analysis to determine significance between groups was performed using GraphPad Prism 9 using a Two-Way ANOVA with a post-hoc tukey’s multiple comparisons test which compared the sample effects within each row.

### Quantitative image analysis of mitochondrial dynamic using kymographs

Cells were plated in an Ibidi 8 well plate at 50, 000 cell/ml. Following co-transfection with GFP-FUS constructs (250 ng) and a Ds-Mitored plasmid (250 ng) at DIV6, cells were incubated for 48 h before imaging on the Nikon Eclipse Ti Spinning disk confocal microscope at 63X. Images were taken every 30 s over 10 min. Channels were split using Image J and a line was drawn along a dendrite (100 μm), to create a kymograph using an Image J plugin. After a kymograph had been created, individual particles were traced using neuron J plugin on Image J to calculate if a particle moved in a retrograde or anterograde direction. Stationary mitochondria were counted if there was no visible movement of that particle in the kymograph. Mitochondrial dynamics were performed on a single dendrite from eight individual cells across three individual experiments per condition. All statistical analysis to determine significance between groups was performed using GraphPad Prism 9 using a One-Way ANOVA with post-hoc tukey’s multiple comparisons test.

### Proximity ligation assay

Proximity ligation assays (PLAs) were performed essentially as the manufacture instructions (Sigma-Aldrich). Briefly, neurons were fixed in 4% paraformaldehyde in PBS and probed with mouse anti-FUS (1:200, Proteintech) and anti-SNPH (1:200, Proteintech), and signals developed using a Duolink In Situ Orange kit (Sigma-Aldrich). Following PLAs, neurons were immunolabeled for chicken anti-MAP2 (1:1000, AbCam). Images were taken at × 60 (oil) on a Nikon Ti-E Two Camera microscope. Images were analysed in ImageJ and positive puncta counted using the cell counting tool. Five somas for each image were analysed for the soma count and 3 different neurites for each of five cells per image were analysed with three biological replicates carried out.

### Puromycin assay

Following transfection of DIV6 neurons as previously described, neurons were treated with 1 mL/well of 1 × ACSF (10 × ACSF with H_2_O, Glucose 11 mM and HEPES 5 mM), MgCl_2_ 1.25 mM and CaCl_2_ mM (pH 7.4) at DIV8. Following a 1-h incubation, 5μL of puromycin (P8833, 10 mg/mL, Sigma Aldrich) was added to each well. After 10 min, cells were fixed and immunostained with mouse anti-puromycin (1:1000, 3RH11, Kerafast) and chicken anti-MAP2 (1:1000, AbCam). Images were taken with a Nikon iSIM super resolution microscope at × 100 (oil) objective. Puromycin puncta were thresholded to be bigger than five pixels in size and average intensity was calculated. Images were obtained from three independent experiments and five different somas and neurites were analysed for each repeat for each condition and values were normalised to threshold. All statistical analysis to determine significance between groups was performed using GraphPad Prism 9 using a One-Way ANOVA with post-hoc tukey’s multiple comparisons test.

### Fish stock maintenance, husbandry and embryo collection

All Danio rerio lines were raised and maintained at 28 °C on a 14 h light/ 10 h dark cycle in the Guy’s Campus Zebrafish facility, London. Embryos were collected and incubated in dishes filled with system water with methylene blue in a 28 °C incubator until experimentation. Morphological staging was used to determine embryo development^46^.

### Microinjection procedure

To deliver the plasmid into individual embryos, 1 mm single capillary needles with filament (world precision instruments) were pulled on a model P-97 flaming/brown micropipette puller (Sutter instrument Co.). Once the micropipette was created, 2.8 μl of plasmid was taken up and attached to manual micromanipulator apparatus. 0.5 nl of plasmid solution (50 ng/μl) was measured on a graticule (Pyser-SGI) and injected into a 1-cell stage embryo using the Picospritzer 111 microinjector (Parker instrumentation).

### UAS: eGFP-FUS constructs

Homologous sticky-end restriction sites were used (PciI and NheI) to allow insertion of the UAS promoter. Initially, both the pN2 5UAS eGFP and the pC1 CMV eGFP-FUS were digested with PciI (NEB). Following digestion, each digested plasmid was purified and then digested with NheI (NEB). After each plasmid had been digested with both enzymes, the UAS insert and eGFP vector were gel extracted, and gel purified (Qiagen) before being ligated and transformed into competent cells (NEB). UAS: eGFP-FUS^WT^ and eGFP-FUS^R514G^ and eGFP-FUS^ΔNLS^ constructs were microinjected at 25 ng/μl along with 25 ng/μl of MNX1:Gal4 plasmid.

### Immunofluorescence

2-day post fertilisation (Long pec) embryos were fixed and, if needed, stained overnight with primary antibody mouse anti-SV2 (1:100, DSHB) followed by overnight staining with alexa-555 conjugated anti-alpha Bungarotoxin (1:100, Invitrogen) and alexa-conjugated goat anti-mouse (1:1000, Invitrogen).

### Morphological analysis of motor neurons

Images were taken on a Nikon Eclipse C1 confocal microscope using a × 40 water objective (N.A. 0.8). Images were based on eGFP expression and z-stacks taken at 1 μm increments. Maximum projection images of the eGFP channel were converted into an 8-bit gray scale image and axons were traced using an available plugin on Image J (Neuron J). After tracing, sholl analysis was performed to assess dendritic complexity from the soma at 10 μm increments. To measure axonal length and branch numbers, axonal branches that were 0.5 μm and larger were included in the axonal branching analysis. Neurons were obtained from six independent experiments and six axons of each condition were chosen. All statistical analysis to determine significance between groups was performed using GraphPad Prism 9 using a One-Way ANOVA with post-hoc tukey’s multiple comparisons test, unless otherwise stated.

### Quantification of synaptic density and colocalisation

Z-sacks images with 1 μm increment which were obtained using a × 40 oil objective (N.A. 1.3) and obtained on the Zeiss Axio Imager Z2 LSM 800 Confocal. Maximum projection images were converted to 8-bit gray-scale in Image J and individual channels were then used to select the threshold which was kept consistent to the control (eGFP) channel. Following imaging, selected axons were extracted and their length was recorded (which allowed calculation of density). Colocalisation analysis was performed as for the rat primary neuron analysis. Analysis was performed on six separate axons from six biological replicates. All statistical analysis to determine significance between groups was performed using GraphPad Prism 9 using a One-Way ANOVA with post-hoc tukey’s multiple comparisons test.

## Supplementary Information

Supplementary Information.

## Data Availability

The datasets used and analysed during the current study are available from the corresponding author on reasonable request. Representative is presented and all data analysed during this study are included in this published article [and its supplementary information files].

## Abbreviations

ALS: Amyotrophic lateral sclerosis
ALS-FUS: ALS-associated FUS
FTD: Frontotemporal dementia
FUS: Fused in sarcoma
NLS: Nuclear localising signal
NMJ: Neuromuscular junction
SNPH: Syntaphilin
SYN: Synaptophysin
PSD-95: Post-synaptic density 95
BTX: Bungarotoxin
SV2: Synaptic vesicle 2
PLA: Proximity ligation assay
