# Supplementary material for: Identification of a novel interaction of FUS and syntaphilin may explain synaptic and mitochondrial abnormalities caused by ALS mutations
Source: Sci Rep. 2021 Jun 30;11:13613. doi: 10.1038/s41598-021-93189-6 (PMC8245466; doi:10.1038/s41598-021-93189-6)
Supplement: Supplementary file 1 — Supplementary Information. [file 41598_2021_93189_MOESM1_ESM.pdf]

Supplementay material associated with

Identification of a novel interaction of FUS and syntaphilin may explain synaptic and mitochondrial abnormalities caused by ALS mutations

Shaakir Salam[1,2], Sara Tacconelli[1], Bradley N Smith[1,2], Jacqueline C Mitchell[1], Elizabeth Glennon[1], Nikolas Nikolaou[2,3], Corinne Houart[2], Caroline Vance[1*]

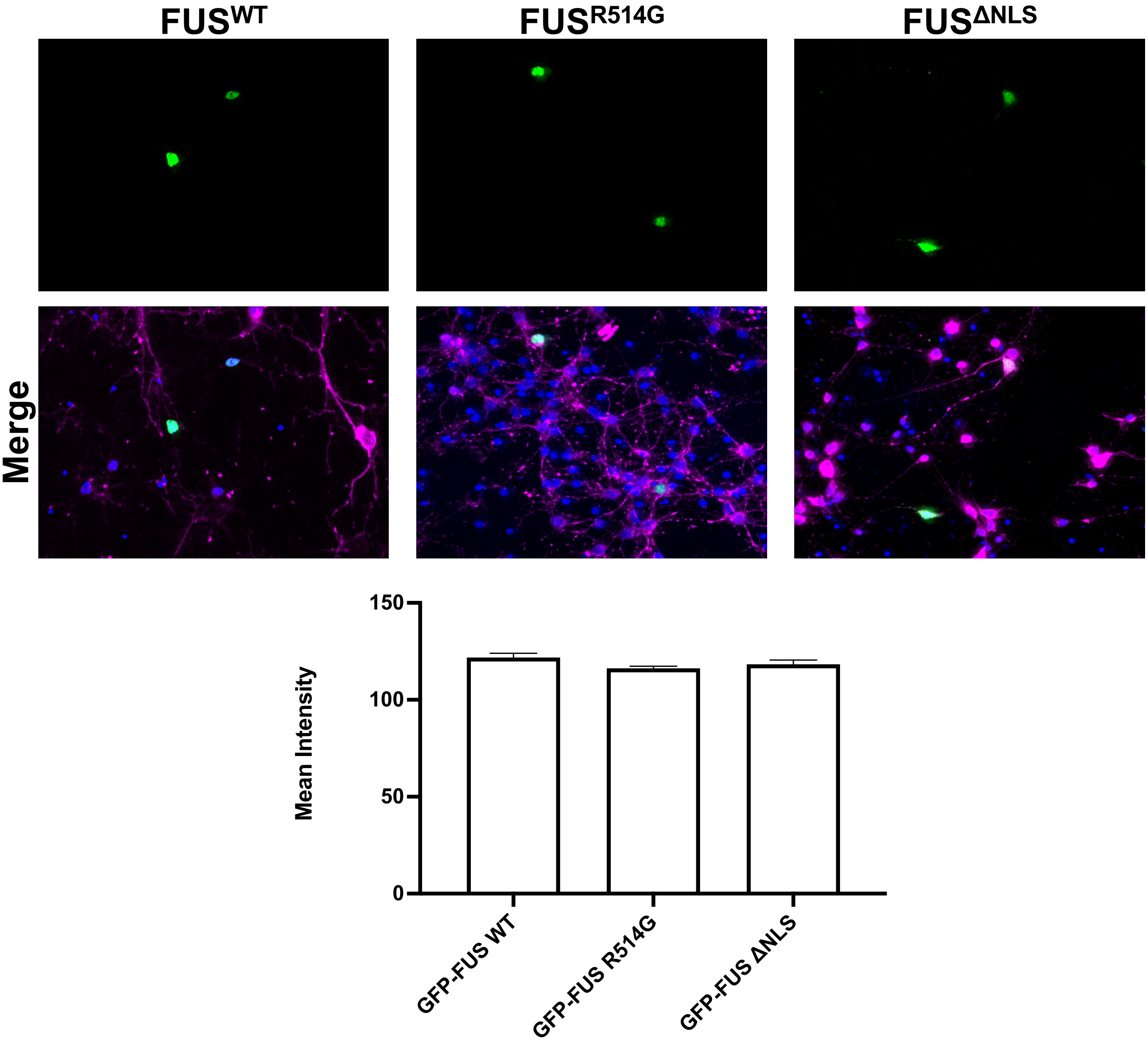

Figure S1: **Quantification of GFP signal across conditions in primary neurons**. Low power images (20X) were taken of primary rat cortical neurons transfected with eGFP-FUS^{WT}, eGFP-FUS^{R514G} and eGFP-FUS$^{\Delta NLS}$. Top panel shows GFP signal only. The merge shows GFP (green), MAP2 (magenta) and nuclei counterstained with DAPI (blue). Quantification of the total GFP signal from 5 representative images of each coverslip was measured and the mean intensity calculated. There were no significant differences in the GFP signal between eGFP-FUS^{WT} and eGFP-FUS^{R514G} (P=0.16) eGFP-FUS^{WT} and eGFP-FUS$^{\Delta NLS}$ (P=0.39) and eGFP-FUS^{R514G} and eGFP-FUS$^{\Delta NLS}$ (p=0.73). Statistical analysis was performed using a one-way ANOVA with a post-hoc Tukey's multiple comparison test.

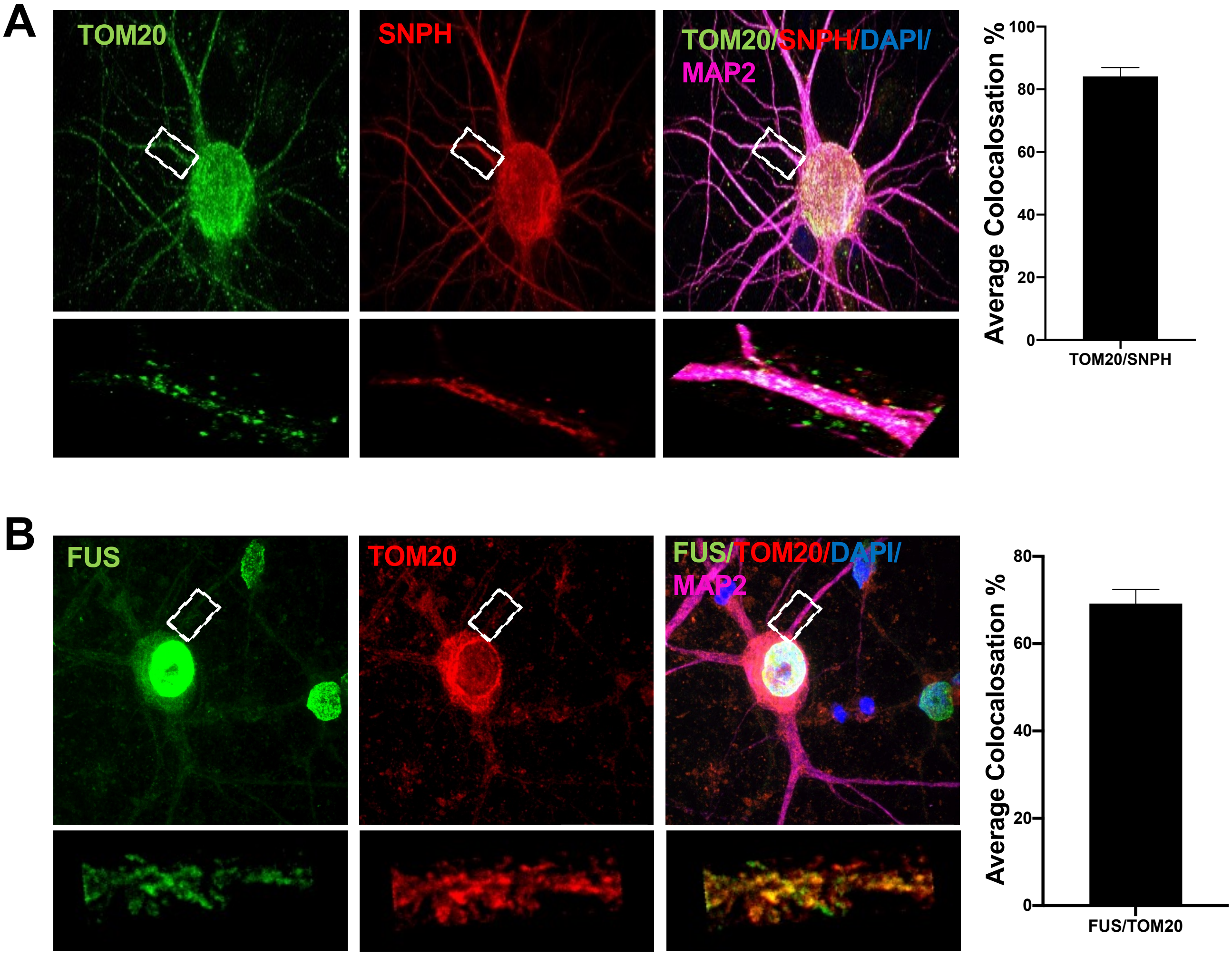

Figure S2: Immunocytochemistry of TOM20 in rat primary neurons: (A) Immunofluorescent staining of DIV21 rat primary cortical neurons. (A) Representative confocal images of SNPH (red) and TOM20 (green) SNPH was found to co-localise with over 80% of TOM20 staining along neurites. (B) Representative confocal images of FUS (green) and the mitochondrial marker TOM20 (Red). FUS was found to localise with almost 70% of TOM20 along neurites. Selected regions of interest for A and B have been shown as magnifications as single and merged channels below the images of the respective neurons with white arrows indicating colocalisation. Nuclei are counterstained blue with DAPI.

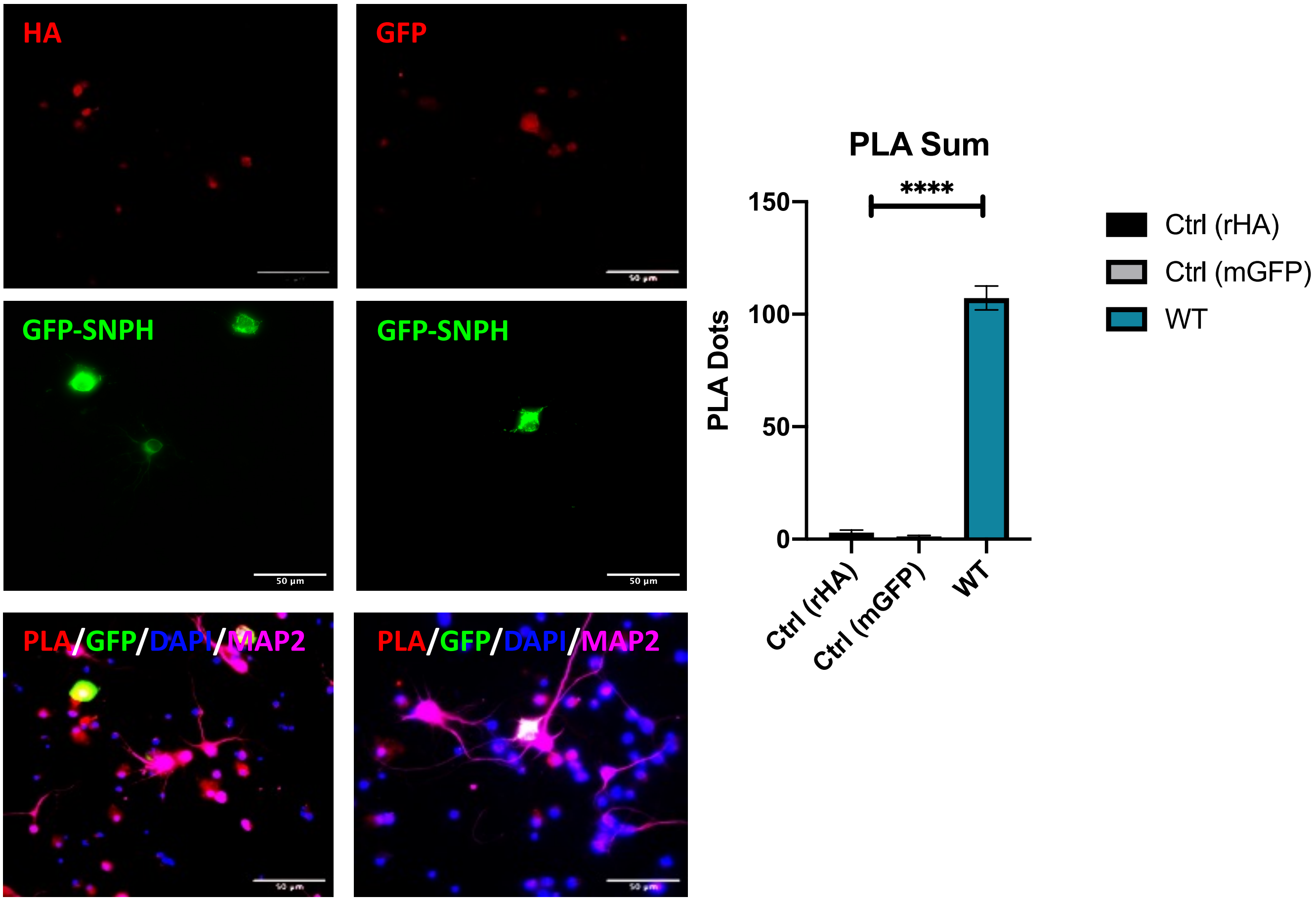

Figure S3: PLA controls for HA and GFP transfected neurons. Single antibody controls for the HA-FUS and GFP-SNPH transfection were carried out whereby only one of the two antibodies was used on double transfected cells and the PLA protocol carried out as normal. Whilst there is some background, there is a significant difference when comparing the number of PLA dots in the single antibody controls to those seen in the HA-FUS^{WT} and GFPSNPH with both antibodies present ($p<0.0001$). Statistical analysis was performed using a one-way ANOVA with a post-hoc Tukey's multiple comparison test.
